# Microemulsion Drug Delivery System: For Bioavailability Enhancement of Ampelopsin

**DOI:** 10.5402/2012/108164

**Published:** 2012-07-08

**Authors:** Shailendra Singh Solanki, Brajesh Sarkar, Rakesh Kumar Dhanwani

**Affiliations:** ^1^Department of Pharmaceutics, College of Pharmacy, IPS Academy, Rajendra Nagar, Indore 452012, India; ^2^Department of Pharmaceutics, Sri Balaji College of Pharmacy, Jaipur 302012, India; ^3^Formulation Research and Development, Dr. Reddy's Laboratories Ltd., Hyderabad 500034, India

## Abstract

Ampelopsin, one of the most common flavonoids, reported to possess numerous pharmacological activities and shows poor aqueous solubility. The purpose of this study was to enhance the dissolution rate and bioavailability of this drug by developing a novel delivery system that is microemulsion (ME) and to study the effect of microemulsion (ME) on the oral bioavailability of ampelopsin. Capmul MCM-based ME formulation with Cremophor EL as surfactant and Transcutol as cosurfactant was developed for oral delivery of ampelopsin. Optimised ME was evaluated for its transparency, viscosity, percentage assay and so forth. Solubilisation capacity of the ME system was also determined. The prepared ME was compared with the pure drug solution and commercially available tablet for *in vitro* drug release. The optimised ME formulation containing ampelopsin, Capmul MCM (5.5%), Cremophor EL (25%), Transcutol P (8.5%), and distilled water showed higher *in vitro* drug release, as compared to plain drug suspension and the suspension of commercially available tablet. These results demonstrate the potential use of ME for improving the bioavailability of poor water soluble compounds, such as ampelopsin.

## 1. Introduction

**Figure 1:**
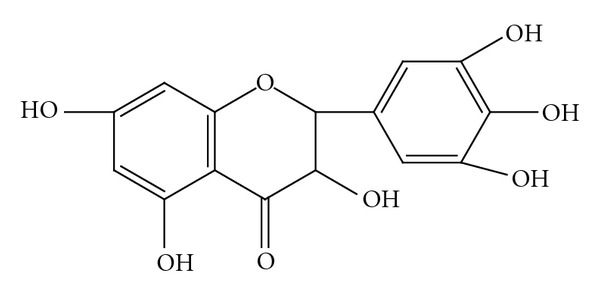
Structure of ampelopsin.

Ampelopsin, isolated from the tender stem and leaves of the plant species *Ampelopsis grossedentata *(Hand-Mazz)   W. T. Wang, was one of the most common flavonoids (Figure 1). Ampelopsin was reported to possess numerous pharmacological activities, such as anti-inflammatory, antimicrobial activity, relieving cough, antioxidation, antihypertension, hepatoprotective effect and anticarcinogenic effect [1–4]. However, the ampelopsin has very less solubility in water (0.2 mg/mL at 25°C), it has very low permeability through intestinal mucosa [5]. Microemulsion (ME), a novel drug delivery system, has been reported to improve the rate and extent of absorption of lipophilic drugs. As a modern drug carrier system, microemulsions are defined as single optically isotropic and thermodynamically stable solution with droplet sizes in the submicron range. In general, they consist of an oil phase, a surfactant, a cosurfactant, and an aqueous phase. Some advantages offered by microemulsions include improvement in drug solubility, enhancement of bioavailability, protection of the drug against the environment, ease of manufacturing, and a long shelf life [6–13]. Use of ampelopsin in most pharmaceutical preparations and some research experiments was thereby limited due to its low water solubility, low intestine permeability and degradation in solution. To our knowledge, no information is available on the improvement of properties of ampelopsin. The main aim of this study was to improve the bioavailability of Ampelopsin by preparing its ME. In the present study, ME formulation was prepared using Capmul MCM (HLB = 5.5–6.0), Cremophor EL (HLB = 14), Transcutol P (HLB = 4) and distilled water by water titration method. The effects of formulation variables on different physicochemical characteristics were studied. An *in vitro* diffusion study was performed using a synthetic membrane. *In vitro* stability of formulation was also assessed.

## 2. Method

### 2.1. Materials

Ampelopsin was obtained from Novopharm Formulations (Pvt. Ltd., Gujarat, India). Capmul MCM, Labrafac CC, Cremophor EL, Labrasol, and Transcutol P were obtained from Colorcon Asia (Mumbai). All other chemicals and reagents were of AR grade. Double-distilled water was used throughout the experiment.

### 2.2. Preparation of ME

ME formulations were prepared by the water titration method by varying the ratio of oil, surfactant, cosurfactant and water; keeping the concentration of Ampelopsin constant in each case (Table 1). The drug was mixed in an accurate quantity of oil (Capmul MCM), and to that, surfactant (Cremophor EL) and cosurfactant (Transcutol P) were added and mixed gently for 10 minutes with the help of a magnetic stirrer at 40 ± 2°C. The mixture was then finally titrated with distilled water until a stable, and transparent ME was obtained. ME formulation was optimized through the formulation (oil : surfactant, surfactant : cosurfactant, and oil : water ratios) and process variables (time and speed). Percentage transmittance was evaluated during the optimization.

### 2.3. Characterisation and Evaluation of ME [14–17]

#### 2.3.1. Visual Inspection

The systems were visually inspected for homogeneity, optical clarity, and fluidity.

#### 2.3.2. Percentage Transmittance

Transparency of both optimized ME formulation and its diluted forms (10 and 100 times with distilled water) was determined by measuring percentage transmittance through ultraviolet spectrophotometer (UV-1601-220x, Shimadzu). Percentage transmittance of samples was measured at 292 nm using purified water as blank.

#### 2.3.3. Examination Under Cross-Polarizing Microscope

Microemulsions were subjected to examination under cross-polarizing microscope for the absence of birefringence to exclude liquid crystalline systems.

#### 2.3.4. Rheological Measurements

The rheological behaviour of ME (0.5 mL) was evaluated using Brookfield LVDV and CP Viscometer (Brookfield, USA) by means of rheological software. Both rheometers were equipped with automatic gap setting. A 5 cm, 18 cone (CP-40), and plate geometry with a solvent trap was used to all investigated samples, and the temperature in the measuring geometry was controlled to within 25 ± 0.1°C by a Peltier system at 10 rpm. All measurements were performed with the instrument in the oscillatory shear mode, and to ensure that all determinations were performed in the linear viscoelastic regime, each rheological determination was preceded by stress-sweep measurements.

#### 2.3.5. Conductivity Study

Electrical conductivity of ME was measured using a conductometer [(CM 180 conductivity meter (Elico, India))]. Calibration was done using freshly prepared standard KCl solutions. To ensure efficient phase separation of excess water, samples at *W*_max⁡_ were left for 24 hours in a water bath (25°C) before the conductivity of the microemulsion was determined. All samples were studied at 25 ± 0.01°C.

#### 2.3.6. Determination of Drug Content in the ME

The loading efficiency of drug in each formulation was determined spectrophotometrically at 292 nm, 5 mL of ME formulation was diluted upto 25 mL with acetonitrile and centrifuged at 4000 rpm at 25 ± 0.01°C for 60 minutes using Eppendorf Centrifuge 5810R. Supernatant was separated and filtered; 1 mL of the supernatant was taken from that solution and diluted to 25 mL of phosphate buffer pH 6.8.

#### 2.3.7. *In vitro* Release Studies

*In vitro *release studies were performed using a modified Franz diffusion cell at 37 ± 2°C. A dialysis membrane, with a pore size of 2.4 nm, was used. Each 1 gm of ME of drug (Ampelopsin), plain drug suspension, and suspension of commercially available tablet of Ampelopsin were placed in the donor compartment. The receptor compartment was filled with dialysis medium (25 mL of phosphate buffer pH 6.8). All samples were studied at 25 ± 0.01°C and 75 rpm (magnetic stirrer). At a fixed time interval of one hour, 5 mL of the sample was withdrawn from the receiver compartment through a side tube and analyzed spectrophotometrically at 292 nm.

## 3. Results and Discussion

### 3.1. Solubility Study

The solubility data of Ampelopsin in various vehicles are provided in Table 2. Capmul MCM showed higher solubilising capacity compared to other vehicles. Hence, Capmul MCM was selected as the oil phase. Cremophor EL and Transcutol P were selected as the surfactant and cosurfactant, respectively for the preparation of optimised ME (Table 3).

### 3.2. Percentage Transmittance

Percentage transmittance of ME after 10 times and 100 times dilution was 98.39% and 98.12%, respectively; indicating transparency and stability of optimized ME.

### 3.3. Examination under Cross-Polarizing Microscope

Examination under cross-polarizing microscope showed dark field indicating no change in isotropic character, and no crystals of the drug were detected, indicating that the drug was completely dissolved and has optical isotropy property.

### 3.4. Determination of Drug Content

Drug content in the optimized ME formulation was found to be 98.11%.

### 3.5. Rheological Study

**Figure 2:**
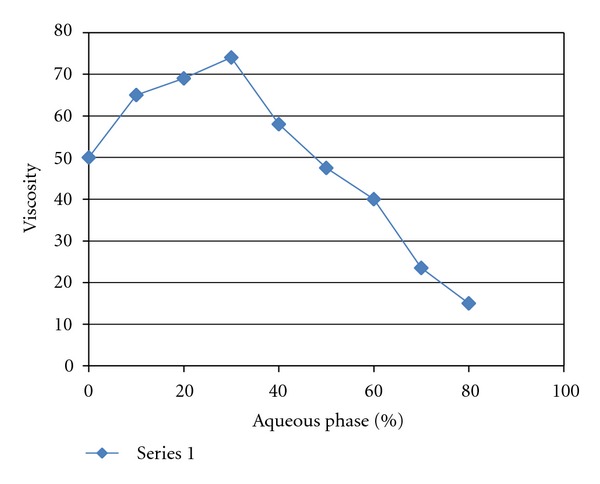
Viscosity changes of ME with increasing water content.

The structure and type of ME system was characterized by rheological measurements. Results obtained from the viscosity study reveal that viscosity increased from 50.31 cP to 75.42 cP, with increasing water content which then gradually decreased (Figure 2). This may be due to the fact that the system transforms from W/O through bicontinuous structure to O/W system.

### 3.6. Electroconductivity Measurement

**Figure 3:**
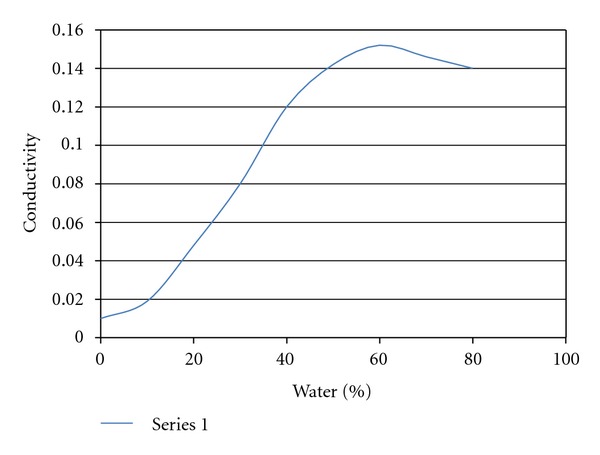
Results of the electroconductivity study.

Results indicated that electrical conductivity increased rapidly up to 59.54% of the aqueous phase addition. Therefore, it was not affected significantly with further addition of the aqueous phase (Figure 3).

### 3.7. *In Vitro* Release of Drug

**Figure 4:**
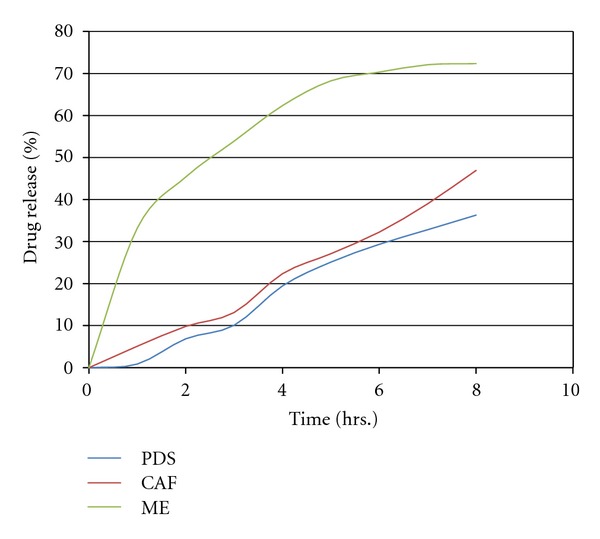
*In vitro *drug release of drug from microemulsion (ME), commercially Ampelopsin formulation (CAF), and plain drug suspension (PDS).

Results of the *in vitro *drug release from the optimized ME, plain drug suspension, and the commercially available drug are shown in Figure 4. Drug release (in hours) from optimized ME, plain drug suspension, and the commercially available drug were found to be 72.34%, 36.28%, and 46.91%, respectively. ME showed higher drug release as compared to plain drug suspension and the commercially available Ampelopsin, which may be due to the solubility-enhancing component of the surfactant and cosurfactant.

## 4. Conclusion

Physicochemical characterization showed that the system undergoes a structural transition from water-in-oil to bicontinuous microemulsion system upon addition of water. The conductivity and viscosity studies provided evidence for the structural transition from water-in-oil to bicontinuous phases. ME-C was found to be the best for use as a drug delivery system on the basis of its optimal solubility for Ampelopsin. After the incorporation of the drug, the microemulsion systems remained stable and optically clear showing no phase separation. The solubility of the drug was confirmed using conductivity measurements which indicated that the drug may be present at the interface of the oil and aqueous phases. UV-visible spectroscopic studies indicated that the system was optically clear. We can conclude that our microemulsion system helps increase the solubility of the hydrophobic drug with the help of hydrophobic component of microemulsion and lipophilic part of the surfactant. The developed ME, containing Capmul MCM (5.5%), Cremophor EL (25%), Transcutol P (8.5%), and distilled water, was found to be a transparent fluid. ME showed higher *in vitro *drug release when compared with plain drug suspension and the suspension of commercially available drug. Hence, on the basis of physicochemical characterization and spectroscopic studies it may be concluded that the ME formulation can be employed to improve the bioavailability of a poorly soluble drug like Ampelopsin. However, further studies on higher animals and humans need to be performed before this formulation can be commercially exploited.

## Figures and Tables

**Table 1:** Content in different ratio for selected microemulsion.

System	Oil	Surfactant	Cosurfactant	Water	Drug
ME-A	1	1	1	3	1
ME-B	3	6	3	10	1
M E-C	5	10	2.5	20	1

**Table 2:** Solubility studies in various vehicles.

Vehicles	Solubility [(mg/mL) ± standard deviation (SD)]
Capmul MCM	51.5 ± 1.6
Labrafac CC	23 ± 2.2
Cremophor EL	24.4 ± 2.7
PEG 600	7.51 ± 0.8
Transcutol P	29.11 ± 1.4
Optimised ME	175.21 ± 2.3

**Table 3:** Content of optimized formulation ME-C.

System	Oil	Surfactant	Cosurfactant	Water	Drug
ME-C	5	10	2.5	20	1

## References

[1] Ye J, Guan Y, Zeng S, Liu D (2008). Ampelopsin prevents apoptosis induced by H_2_O_2_ in MT-4 lymphocytes. *Planta Medica*.

[2] TAN GY, ZHANG MH, FENG JH, HAN AY, ZHENG SS, XIE P (2010). Effects of pretreatment by the flavanol ampelopsin on porcine kidney epithelial cell injury induced by hydrogen peroxide. *Agricultural Sciences in China*.

[3] Qi S, Xin Y, Guo Y (2012). Ampelopsin reduces endotoxic inflammation via repressing ROS-mediated activation of PI3K/Akt/NF-*κ*B signaling pathways. *International Immunopharmacology*.

[4] Kundaković T, Stanojković T, Milenković M (2008). Cytotoxic, antioxidant, and antimicrobial activities of Ampelopsis brevipedunculata and Parthenocissus tricuspidata (Vitaceae). *Archives of Biological Sciences*.

[5] Ruan LP, Yu BY, Fu GM, Zhu DN (2005). Improving the solubility of ampelopsin by solid dispersions and inclusion complexes. *Journal of Pharmaceutical and Biomedical Analysis*.

[6] Ajazuddin, Saraf S (2010). Applications of novel drug delivery system for herbal formulations. *Fitoterapia*.

[7] Dong X, Ke X, Liao Z (2011). The microstructure characterization of meloxicam microemulsion and its influence on the solubilization capacity. *Drug Development and Industrial Pharmacy*.

[8] Malcolmson C, Lawrence MJ (1995). Three-component non-ionic oil-in-water microemulsions using polyoxyethylene ether surfactants. *Colloids and Surfaces B*.

[9] Constantinides PP, Scalart JP, Lancaster C (1994). Formulation and intestinal absorption enhancement evaluation of water-in-oil microemulsions incorporating medium-chain glycerides. *Pharmaceutical Research*.

[10] Lopez F, Cinelli G, Ambrosone L, Colafemmina G, Ceglie A, Palazzo G (2004). Role of the cosurfactant in water-in-oil microemulsion: interfacial properties tune the enzymatic activity of lipase. *Colloids and Surfaces A*.

[11] Brime B, Moreno MA, Frutos G, Ballesteros P, Frutos P (2002). Amphotericin B in oil-water lecithin-based microemulsions: formulation and toxicity evaluation. *Journal of Pharmaceutical Sciences*.

[12] Badawi AA, Nour SA, Sakran WS, El-Mancy SMS (2009). Preparation and evaluation of microemulsion systems containing salicylic acid. *AAPS PharmSciTech*.

[13] Ghosh PK, Majithiya RJ, Umrethia ML, Murthy RSR (2006). Design and development of microemulsion drug delivery system of acyclovir for improvement of oral bioavailability. *AAPS PharmSciTech*.

[14] Singh AK, Chaurasiya A, Awasthi A (2009). Oral bioavailability enhancement of exemestane from self-microemulsifying drug delivery system (SMEDDS). *AAPS PharmSciTech*.

[15] Yang JG, Liu BG, Liang GZ, Ning ZX (2009). Structure-activity relationship of flavonoids active against lard oil oxidation based on quantum chemical analysis. *Molecules*.

[16] Zhang YS, Ning ZX, Yang SZ, Wu H (2003). Antioxidation properties and mechanism of action of dihydromyricetin from Ampelopsis grossedentata. *Yaoxue Xuebao*.

[17] Li X, Yuan Q, Huang Y, Zhou Y, Liu Y (2010). Development of silymarin self-microemulsifying drug delivery system with enhanced oral bioavailability. *AAPS PharmSciTech*.

